# Dissociable Roles of Dorsolateral Prefrontal Cortex and Frontal Eye Fields During Saccadic Eye Movements

**DOI:** 10.3389/fnhum.2015.00613

**Published:** 2015-11-16

**Authors:** Ian G. M. Cameron, Justin M. Riddle, Mark D’Esposito

**Affiliations:** ^1^Helen Wills Neuroscience Institute, University of California, BerkeleyBerkeley, CA, USA; ^2^Department of Psychology, University of California, BerkeleyBerkeley, CA, USA

**Keywords:** anti-saccade, executive control, cTBS, transcranial magnetic stimulation (TMS), oculomotor, FEF, DLPFC

## Abstract

The dorsolateral prefrontal cortex (DLPFC) and the frontal eye fields (FEF) have both been implicated in the executive control of saccades, yet possible dissociable roles of each region have not been established. Specifically, both establishing a “*task set*” as well as suppressing an inappropriate response have been linked to DLPFC and FEF activity, with behavioral outcome measures of these mechanisms mainly being the percentage of pro-saccade errors made on anti-saccade trials. We used continuous theta-burst stimulation (cTBS) to disrupt FEF or DLPFC function in humans during an anti-saccade task to assess the causal role of these regions in these executive control processes, and in programming saccades towards (pro-saccade) or away (anti-saccade) from visual targets. After right FEF cTBS, as compared to control cTBS to the right primary somatosensory cortex (rS1), anti-saccade amplitude of the first saccade decreased and the number of anti-saccades to acquire final position increased; however direction errors to the visual target were not different. In contrast, after left DLPFC cTBS, as compared to left S1 cTBS, subjects displayed greater direction errors for contralateral anti-saccades; however, there were no impairments on the number of saccades or the saccade amplitude. These results are consistent with the notion that DLPFC is necessary for executive control of saccades, whereas FEF is necessary for visuo-motor aspects of anti-saccade programming.

## Introduction

Both the frontal eye fields (FEF) and the dorsolateral prefrontal cortex (DLPFC) are proposed be involved in executive control during anti-saccade tasks (look away from a visual stimulus; Munoz and Everling, 2004). Here, executive control refers to the establishment of a task set, and the suppression against a more automatic pro-saccade response. There are theoretical bases from neurophysiology, lesion studies, and neuroimaging to suggest that both regions are involved in executive control. However, while the findings are more compelling for a critical role of DLPFC, studies have not converged on consistent evidence to support a critical role of FEF.

In FEF, during the preparatory phase of an anti-saccade task (compared to a pro-saccade task), saccade neurons show decreased activity, whereas fixation neurons exhibit increased activity(Everling and Munoz, 2000; Munoz and Everling, 2004), suggesting that a hallmark of anti-saccade task set can be identified in FEF neurons. Patients with frontal lobe lesions encompassing FEF display difficulty in suppressing saccades to visual stimuli (Guitton et al., 1985; Van der Stigchel et al., 2012), suggesting FEF may be important to saccade suppression. Indeed, when subjects must stop a planned saccade from being executed in a stop-signal paradigm, computational models and neurophysiological data support a role for fixation neurons in suppression (Boucher et al., 2007; Schall and Godlove, 2012). However, in a study of a patient with a more circumscribed FEF lesion (Gaymard et al., 1999), and in studies that disrupted FEF function with transcranial magnetic stimulation (TMS; Müri et al., 1991; Nagel et al., 2008), subjects did not exhibit increased pro-saccade errors on anti-saccade trials. Thus, the FEF may be involved in executive control processes during anti-saccade tasks, but whether it is necessary for this function has not been established. It could be that other brain regions, such as the DLPFC, could provide signals that influenceFEF neurons.

DLPFC neurons also exhibit activity consistent with task set signals during the preparatory phase of anti-saccade tasks (Everling and DeSouza, 2005; Johnston and Everling, 2006). Moreover, application of a TMS pulse to DLPFC during the preparatory phase, and not after, results in increased errors in an anti-saccade task (Nyffeler et al., 2007). Also, patients with DLPFC lesions exhibit increased pro-saccade errors on anti-saccade trials (Guitton et al., 1985; Pierrot-Deseilligny et al., 1991, 2003; Ploner et al., 2005), suggesting a role in suppression. Finally, given the strong evidence for a role of the DLPFC in executive functions (Gazzaley and D’Esposito, 2007), it is likely that this region is critical during anti-saccade tasks.

Here, using continuous theta-burst stimulation (cTBS), we disrupted FEF and DLPFC function during the performance of an anti-saccade task. In cTBS, 50 Hz pulse triplets are applied continuously at a 5 Hz frequency for a duration typically of 20–40 s (Huang et al., 2005). It is a method of “offline” TMS, such that the purpose is to modulate brain function and hence subsequent behavioral and neuroimaging measures. While the mechanisms are not fully understood, it is known that cTBS reduces motor cortex excitability, and therefore, it is hypothesized to work via the induction of long term depression (LTD) in cortical synapses (Ziemann and Siebner, 2008; Di Lazzaro et al., 2010). cTBS is a more recent form of repetitive TMS, and is utilized because it is more efficient: 40 s of cTBS can produce inhibitory effects of upwards of 60 min, whereas typical low frequency (1 Hz) repetitive TMS protocols produce effects on the order of 20–30 min following 25 min of application (Touge et al., 2001; Quartarone et al., 2006; Ziemann et al., 2008).

Following cTBS application to the right FEF and the left DLPFC, we were able to assess the effects on saccade behavior when these regions were inhibited. Due to potential non-specific neurostimulation effects, or placebo effects, in each case we compared the behavior to cTBS to the primary somatosensory cortex in the same hemisphere. Our findings support dissociable roles for these two brain regions, such that DLPFC is critical to executive control, but FEF is critical to the visuo-motor aspects of anti-saccade programming (Bruce and Goldberg, 1985; Schall, 2002; Moon et al., 2007).

## Materials and Methods

### General Procedures

We compared the effects of cTBS to right FEF, and to left DLPFC, both compared to cTBS to control regions in the same hemisphere (somatosensory cortex). These studies were performed in two groups of human subjects in an magnetic resonance imaging (MRI) environment, as we wished to examine changes in neural activation related to the cTBS effects in a companion study. The functional MRI (fMRI)/MRI method also allowed us to precisely localize the cTBS targets for every subject.

In the first session subjects underwent functional MRI scanning (to provide functional and anatomical locations of the regions of interest). Right FEF was chosen because the majority of previous TMS studies on FEF used the right hemisphere (Nyffeler et al., 2006; Nyffeler et al., 2008a, b; Van Ettinger-Veenstra et al., 2009; Jaun-Frutiger et al., 2013). Additionally, right FEF may have a more bilateral role in visual processing or attentional control (Grosbras and Paus, 2003; Ruff et al., 2009), thus we wanted to reduce the possibility that any absent effects from cTBS could be explained by the bilateral role of the right hemisphere. In comparison, lesion or TMS studies in the oculomotor system have not revealed systematic behavioral differences between right and left DLPFC disruption (despite a dominance of studying the right hemisphere; Muri et al., 2000; Nyffeler et al., 2002, 2004). However, left DLPFC was chosen because previous studies have revealed greater deficits in task switching after left compared to right DLPFC cTBS (Ko et al., 2008), and also correspondingly, reduced dopamine release in the striatum after left but not right DLPFC cTBS (Ko et al., 2008), but increased dopamine release after left but not right DLPFC 10 Hz rTMS (Cho and Strafella, 2009). It has also been shown that patients with left prefrontal lesions, particularly the dorsolateral portions, display deficits in task set establishment (Stuss and Alexander, 2007; Stuss, 2011).

Eighteen right-handed subjects participated in the FEF study and 19 right-handed subjects participated in the DLPFC study. In the FEF study, two subjects were excluded for not being able to participate in all three required sessions, resulting in six female and ten male participants (mean age of 20.2 ± 1.4 years). Additionally, two subjects (1 male, 1 female) were partially excluded from full analysis for problems on one of the days with eye-tracking illumination or syncing the eye-tracker with the scanner. In these instances, we did not remove subjects where there were problems affecting some behavioral parameters (e.g., reaction time) but not others (e.g., saccade amplitude). The reported degrees of freedom reflect these situations. In the DLPFC study, two subjects withdrew partway through the study, and two subjects were excluded from analysis because they displayed >60% errors, resulting in six females and nine males (mean age 20.7 ± 1.7 years). All subjects were recruited from the student population at UC Berkeley and all had normal or corrected to normal vision. Both studies were approved by the Committee for the Protection of Human Subjects at the University of California, Berkeley. All subjects gave written informed consent in accordance with the Declaration of Helsinki.

### Task Design

**Figure 1:**
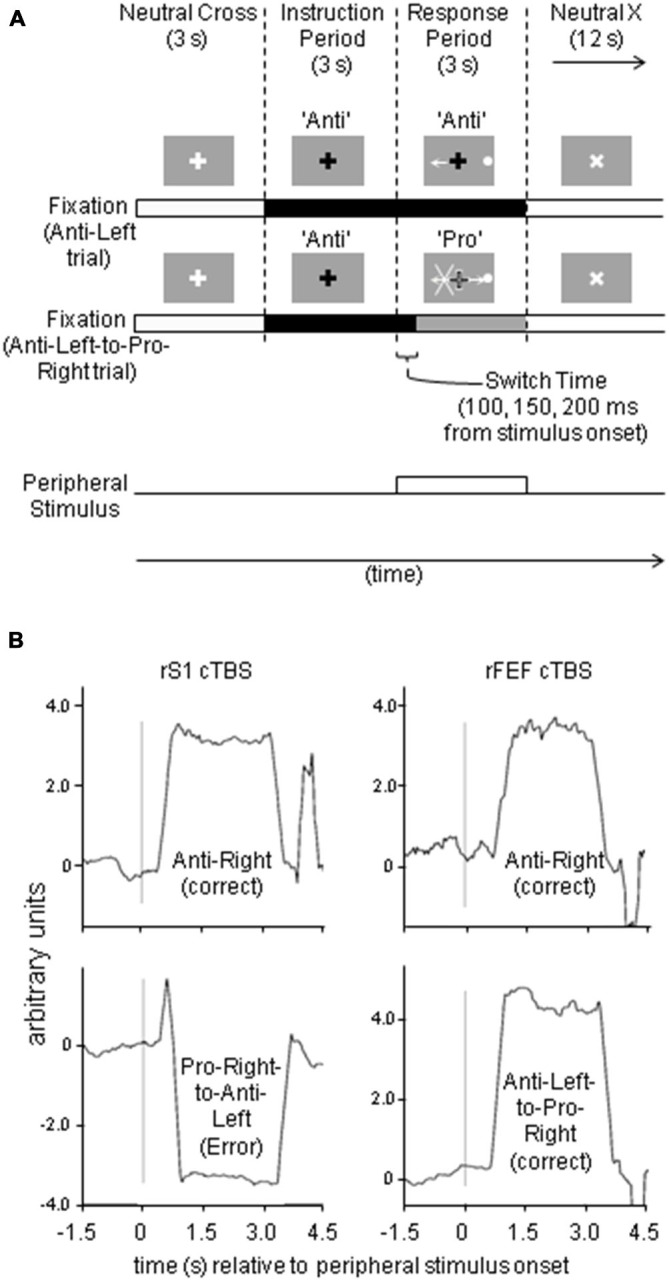
**(A)** Paradigm and stimuli timing, illustrating representative anti-left and anti-left-to-pro-right trials, with the peripheral stimulus on the right. **(B)** Sample eye traces from FEF study.

Twenty trials were presented in a given run (totaling 7 min) following the basic design of Cameron et al. (2009). Subjects were required to make a saccade to a blue disk located in the periphery of the screen, based on a colored fixation instruction (Figure 1A). The peripheral target stimuli were 15^°^ from fixation in the FEF study and 12^°^ from fixation in the DLPFC study. All stimuli were 0.5^°^ in visual angle and same approximate luminance.

Each run contained a predefined pseudorandom presentation of 4 “pro” trials (2 with leftwards target stimulus, 2 with rightwards), 4 “anti” trials, 6 “pro-to-anti” switch trials (from pro- to anti-saccade), and 6 “anti-to-pro” trials. Each trial began with fixation on a blue cross (“neutral cross”) at the center for 3 s that did not convey any saccade instruction. The cross then changed to green (instructing a pro-saccade) or red (instructing an anti-saccade) for 3 s. Next, a blue disk appeared in the periphery and remained illuminated for 3 s (response period). On pro- and anti-saccade trials, participants were instructed to make a saccade to this blue disk, or to its mirror location and to hold their gaze there for 3 s, until another neutral fixation stimulus (“neutral X”) appeared at center for 12 s instructing participants to return their gaze to center. However, on pro-to-anti and anti-to-pro trials, the initial fixation instruction (red or green) switched color at 100, 150 or 200 ms following onset of the peripheral blue disk. Participants were told that if this occurred, they were to obey only the new instruction, and to be as quick and accurate as possible. In all cases, they were told to correct their mistakes.

**Figure 2:**
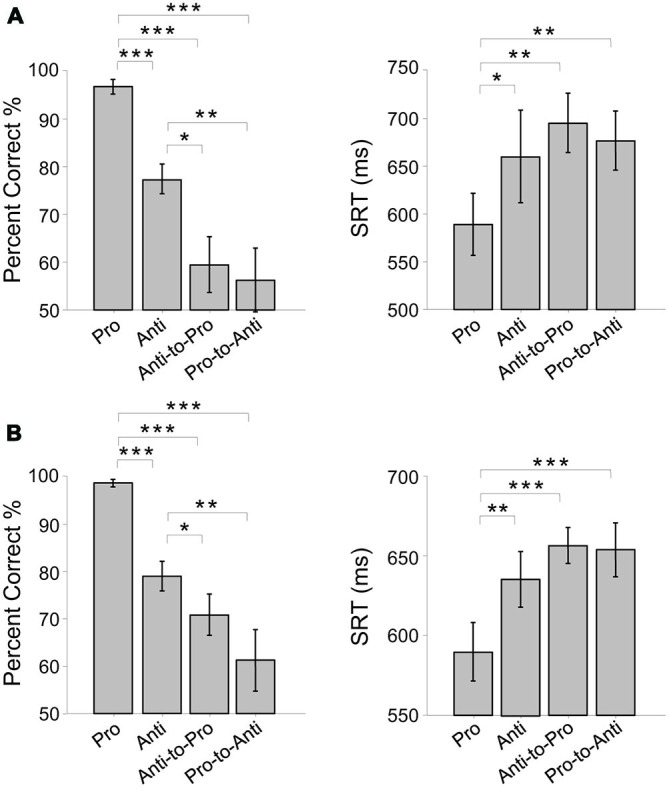
**Correct performance and saccade reaction times (SRT) for pro-, anti-, anti-to-pro and pro-to-anti trials (collapsed across direction) on the first sessions in **(A)** rFEF study **(B)** lDLPFC study.** *p < 0.05, **p < 0.01, ***p < 0.001.

We utilized this task switching design because normal anti-saccade trials cannot dissociate deficits in task set establishment from deficits in suppressing response. If we consider that typical anti-saccade trials (like those of the non-switch condition in the present experiment) require subjects to plan for an anti-saccade during the preparatory period, then they are an example of a behavior that requires executive control in the context of a “task set”. A component of this task set could be to suppress a saccade response to a visual stimulus when it appears. However, as outlined in the introduction, there is evidence for a role of FEF particularly in executive control during a stop-signal task, where subjects do not have a preparatory cue informing them to stop a response. In stop-signal or go/no-go tasks, subjects suppress a prepared response when instructed by a cue that appears after a response is prepared. Thus, the inclusion of the switch trials allows us to explicitly test cases where suppressing a prepared response (but also reconfiguring task set) is required. Switch time variation was used to prevent temporal predictability, and previous work has demonstrated that a 200 ms switch time is within a critical time period for producing switching costs, signifying that an initial response had been in preparation (Nakamura et al., 2005; Cameron et al., 2007, 2009). We chose the percentage of switch trials (60%) in order to increase their frequency given that switch trials were expected to produce more errors than non-switch trials. Our previous study demonstrated that switch costs are produced with switch trials up to 75% in probability, demonstrating that switch trial predictability cannot override the tendency to prepare the instructed response automatically (Cameron et al., 2007). Importantly, the purpose of the switch trials was not to explicitly examine switch costs, but to examine executive control in situations where subjects must suppress and override a planned response suddenly, and we use this design as a basis for examining differences in this type of task switching under the impact of FEF or DLPFC disruption of function. We verified that the typical behavioral patterns in the switching paradigm were produced in each study (FEF study: Figure 2A; DLPFC study: Figure 2B), by assessing the mean pro-, anti-, anti-to-pro and pro-to-anti percentage correct and saccade reaction times (SRT). As shown in Figure 2; subjects exhibited costs to performing an anti-saccade compared to a pro-saccade and a cost of switching their initial response to the opposite one. Note that indeed the reaction times are longer than typically observed in simpler pro-/anti-saccade studies (on the order of 150–300 ms), which indicates a waiting strategy in the subjects, but which however, does not alter the relative difference in automaticity between pro- and anti-saccades, and the costs associated with switching task.

### TMS Procedures

Three TMS sessions were performed, with subjects performing the same task on each session. On the first session, they did not receive cTBS, but did receive single pulse TMS over left M1 to determine their active motor threshold (AMT) of their dominant (right) hand. The first day was also used to obtain a T1-weighted anatomical MRI scan and to define right FEF or left DLPFC based on performing the task (described in *fMRI Scanning*), counterbalanced with the control site (primary somatosensory cortex) on days 2 and 3. These days were one to two weeks apart at the same approximate time of day. On both days 2 and 3, the AMT procedure was also conducted to confirm the results from day 1. We chose S1 in the same hemisphere as the FEF or DLPFC site to examine the specific effects of cTBS on the saccade network, rather than a placebo effect from sham stimulation, or vertex stimulation, which typically falls between the cerebral hemispheres. The specific rS1 location was the most superior extent of the postcentral gyrus located anatomically on each subjects anatomical scan. This medial position was chosen to avoid stimulating a proprioceptive eye representation of orbital position, which has recently been identified in the lateral depths of the central sulcus in monkeys (Zhang et al., 2008) and humans (Balslev et al., 2011).

cTBS parameters were identical to those described by Huang et al. (2005); consisting of 50 Hz triplets (three single pulses separated by 20 ms) repeated at 5 Hz (every 200 ms) over a period of 40 s (600 pulses totals). This protocol was shown to reduce the motor evoked potential (MEP) for up to 60 min (Huang et al., 2005). A previous study, assessing cTBS in the theta-frequency range (30 Hz triplets at 6 Hz) to FEF, showed that saccade latencies (bi-directionally) were increased for a period of 30 min in three subjects. However, the same protocol showed that cTBS affected fMRI BOLD signal in FEF and elsewhere in the oculomotor network for periods up to 60 min (Hubl et al., 2008).

### Additional Details on FEF TMS Study

Subjects were seated in a comfortable desk chair. To establish resting and AMT, electromyography was recorded using electrodes placed on the first dorsal interosseus (FDI) muscle of their right hand. TMS was applied using a hand-held bi-phasic figure-eight coil with a 70 mm outer winding diameter (Magstim, Whitland, UK) to the left primary motor cortex. First, single pulses were delivered over left M1 defined by the scalp location where TMS produced the largest MEP from the right FDI muscle when the subject’s hand was at rest. Next, AMT was defined as the minimum pulse intensity required to produce an MEP on 5 out of 10 trials when the participant was maintaining a voluntary contraction of their FDI at approximately 20% of maximum. To help the participant maintain a 20% of max contraction, the raw EMG signal recorded from the FDI was displayed on a screen. Stimulation intensity for cTBS was set at 80% of the AMT. TMS coil alignment with rFEF and rS1 was achieved using Brainsight v1.7 (Rogue Research, Montreal, Canada), and the anatomical scan acquired on the first day.

Coil position was chosen to induce lateromedial current flow (45^°^ from the mid-sagittal; O’Shea et al., 2007), but also to provide the maximum operator controlled precision of stimulation with the given experimental setup, which resulted in the coil being positioned at approximately 25^°^ from the sagittal axis, with the handle pointing backwards and across the sagittal axis. We maintained the same orientation between rS1 and rFEF to control for the possibility that one region could have been stimulated by the other (i.e., no part of the figure-eight coil overlapped the other region). It is unlikely that the focality of TMS can be any less than 100–200 mm^2^ (a radius of 6–8 mm; Wagner et al., 2009). Thus, we kept the orientation identical given that the two sites were separated by approximately 30–35 mm, and simply shifted the coil forward (rFEF) or back (rS1) at the same angle. The average coordinates (X Y Z mm, MNI space) of rFEF were 30 ± 6, −6 ± 4, 56 ± 6 and the average coordinates for rS1 were 9 ± 2, −39 ± 5, 79 ± 1. Assuming such a radius of TMS effects at approximately 6–8 mm from the center of the stimulation site, this results in effects at rS1 that are adequately separated from the rFEF site.

### Additional Details on the DLPFC TMS Study

This study employed the same general procedures as in the FEF study. However, subjects were seated in a Gen 3 TMS Chair (Rogue Research, Montreal, Canada), and TMS was applied using an arm-supported Air Film Coil (Magstim, Whitland, UK). For cTBS, the coil was positioned tangentially to the skull surface above the lDLFPC site with the handle pointed backwards at a 45º angle. LDLPFC was localized individually in the middle frontal gyrus (based on task activation), such that the average coordinates (*X* *Y* *Z* mm, MNI space) were: −38 ± 5, 40 ± 4, 28 ± 6. The average LS1 coordinates were −8 ± 2, −43 ± 3, 77 ± 2.

### Visual Stimuli and Display

Visual stimuli were generated in MATLAB using Psychtoolbox running on a Mac, and an AVOTEC video projector was used to back-project the image onto the screen inside the bore placed 36 cm from the mirror. The projector had a refresh rate of 60 Hz and a spatial resolution of approximately 0.15^°^ of visual arc.

### Eye Tracking

Eye position data was recorded at 60 Hz using an infrared AVOTEC camera (Stuart, FL, USA) and Viewpoint software v. 2009b running on a PC (Arrington Research Inc., Scottsdale, AZ, USA). The camera was fixed to the mirror on the MRI head coil, and illuminated the subject’s right eye. The surface of the mirror was ~12.5 cm from each subject’s eyes. Since calibration of the eye tracker was not possible due to the time-sensitive nature of cTBS, only raw eye position output was utilized (see sample eye-trace, Figure 1B). The eye-tracker calibration required approximately 10 min in order to achieve a stable 9- or 16-point calibration. Additionally, frequent re-calibration throughout an experimental session was noted in pilot studies. It was therefore impossible to perform the calibration routine in these time-sensitive cTBS experiment. Note that we used a head coil-fixed eye tracker, so the distance of the subject’s eye from the camera was approximately equal on each day. We then scaled all rightwards or leftwards saccades to the mean pro-saccade amplitudes of the final saccade position in the same direction on day 1.

### Functional MRI Scanning

All MRI scans were conducted at the Henry H. Wheeler Jr. Brain Imaging Center with a Siemens 3T Magnetom Trio system (Erlangen, Germany), with a 12-channel receive-only head coil. A Siemens auto-align scout (45 s) followed by a 3-plane localizer (15 s) were acquired initially; next, six functional runs (each 7 min) were acquired successively; following this, one 5 min resting-state scan was acquired, (but the purpose was not for this study, so will not be described further); finally, an magnetization prepared rapid gradient-echo (MP-RAGE) anatomical scan (5 min) was acquired. Functional scans were collected using a T2^*^-weighted single-shot echo-planar imaging sequence, with slices acquired at 30^°^ to the transverse orientation, and with an anterior/posterior phase-encoding direction. A Siemens Auto-Align scout was employed to preset the location of imaging volume on a three-plane localizer collected initially. For 12/16 subjects in the FEF study, and for all subjects in the DLPFC study, each functional volume contained 32 slices that were 3.3 × 3.3 mm, with a slice thickness of 3.5 mm. A gap of 15% (0.525 mm) was also employed, resulting in a total slice spacing of 4.025 mm. An ascending slice acquisition sequence was used. The repetition time (TR) was 2.0 s, the field of view was 211 mm × 211 mm, and the matrix size was 64 × 64. The flip angle was 77^°^ and the echo time (TE) was 30 ms, in order to optimize for the sensitivity of the BOLD contrast. Fat suppression was used. In the remaining 4/16 subjects, day 1 used a scanning protocol (41 slices, 3.3 mm × 3.3 mm, with a slice thickness of 3.3 mm and no slice gap) that was then changed to the above. On the first trial of every run two non-recorded Siemens “dummy scans” and two additional scans that were also subsequently discarded to achieve steady-state longitudinal magnetization. Parallel imaging (e.g., GRAPPA) was not used. The high-resolution anatomical images were collected with a T1-weighted MPRAGE sequence, with an anterior/posterior phase-encoding direction. The voxel size was 1 mm in all three directions. The field of view was 240 × 256 mm, the flip angle was 9^°^, the TE was 2.98 ms, and the TR was 2300 ms.

### Data Analysis

Eye movement data was analyzed with custom MATLAB v7.11 programs (The MathWorks Inc., Natick, MA, USA) and imaging data were analyzed using BrainVoyager v2.3 (Brain Innovation, Maastricht, The Netherlands). Valid trials, which included correctly executed trials, as well as direction error trials, were first separated from invalid trials, which comprised of: trials with *SRTs* <90 ms (anticipatory errors), trials with SRTs slower than 1200 ms (>3 SD of the mean), saccades made in the wrong direction after a correct response, and saccades during preparatory and fixation periods. Percent correct was then determined using the correctly executed trials and the direction error trials.

There were four parameters of interest used to describe saccade behavior. The first two: percentage correct (based on pro- or anti-saccade instruction) and SRT are typical parameters used to describe, in part, executive control over saccade initiation. We also defined the number of saccades or “steps” taken to reach final position: more than one indicates that the initial saccade program was incorrect. Finally, we examined the saccade amplitude to characterize the metrics of the first saccade made (the first of the steps, or the saccade to final position if no steps were made).

**Figure 3:**
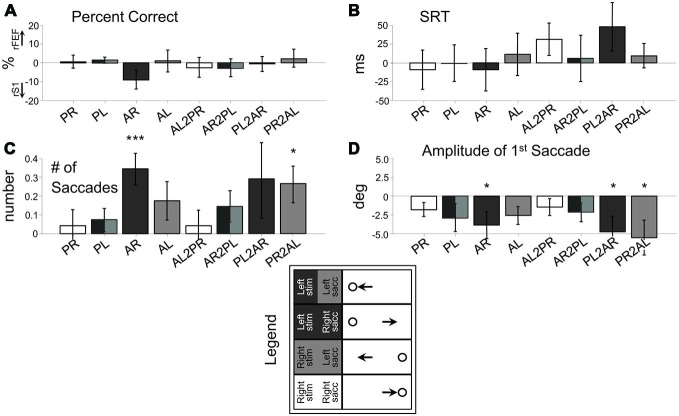
**Behavior difference between rFEF cTBS and rS1 cTBS (rFEF minus rS1) across the eight response types. (A)** Percent Correct. **(B)** Saccade Reaction Time (SRT). **(C)** Number of saccades to reach final position. **(D)** Amplitude of first saccade. Legend categorizes the trial types into four categories (shaded) based on the location of the stimulus (Right and Left) and the direction of the saccade (Right and Left). PR, pro-right; PL, pro-left; AR, anti-right; AL, anti-left; AL2PR, anti-left-to-pro-right; AR2PL, anti-right-to-pro-left; PL2AR, pro-left-to-anti-right; PR2AL, pro-right-to-anti-left. Only correctly performed trials were included. *p < 0.05, **p < 0.01, ***p < 0.001, one-sample *t*-test.

**Figure 4:**
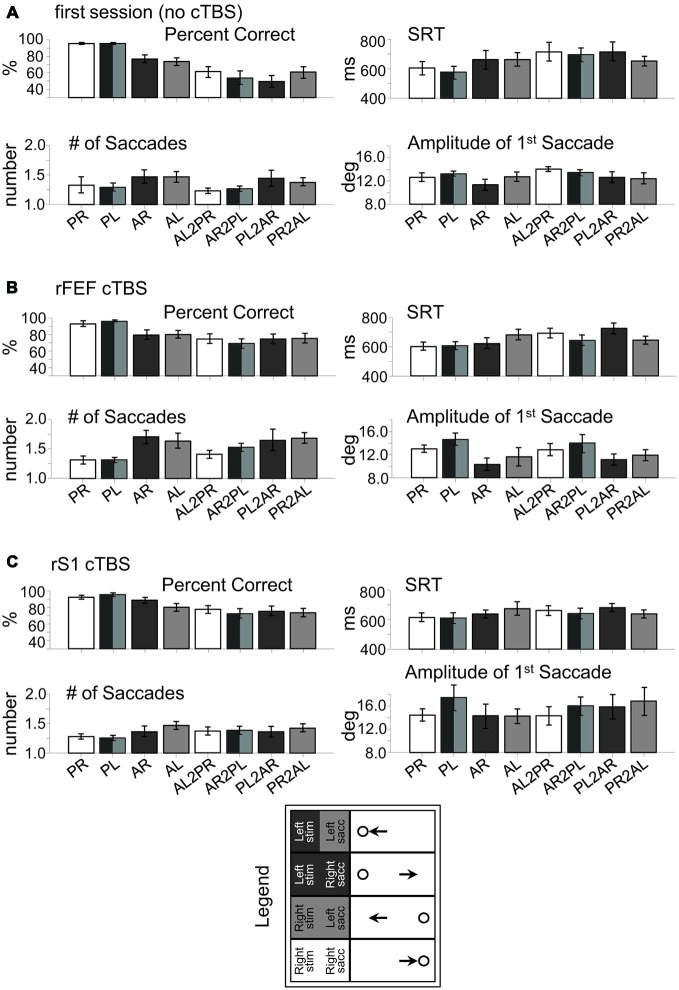
**Raw behavioral data in rFEF study. (A)** Session 1 (no cTBS). **(B)** rFEF cTBS. **(C)** rS1 cTBS. PR, pro-right; PL, pro-left; AR, anti-right; AL, anti-left; AL2PR, anti-left-to-pro-right; AR2PL, anti-right-to-pro-left; PL2AR, pro-left-to-anti-right; PR2AL, pro-right-to-anti-left.

**Figure 5:**
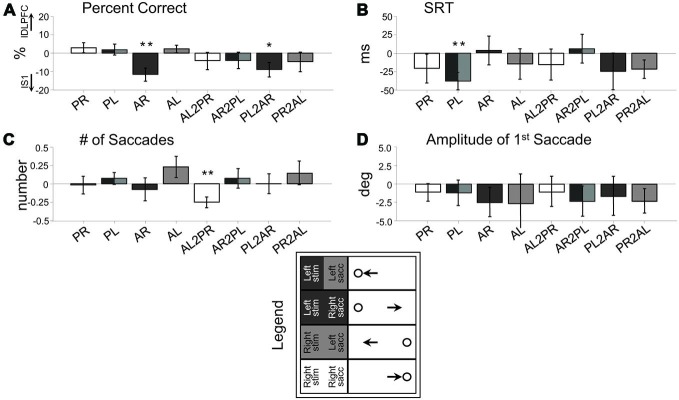
**Behavior difference between lDLPFC cTBS and lS1 cTBS (lDLPFC minus lS1) across the eight response types. (A)** Percent Correct. **(B)** Saccade Reaction Time (SRT). **(C)** Number of saccades to final position. **(D)** Amplitude of first saccade. Conventions are as in Figure 3. *p < 0.05, **p < 0.01, one-sample *t*-test.

**Figure 6:**
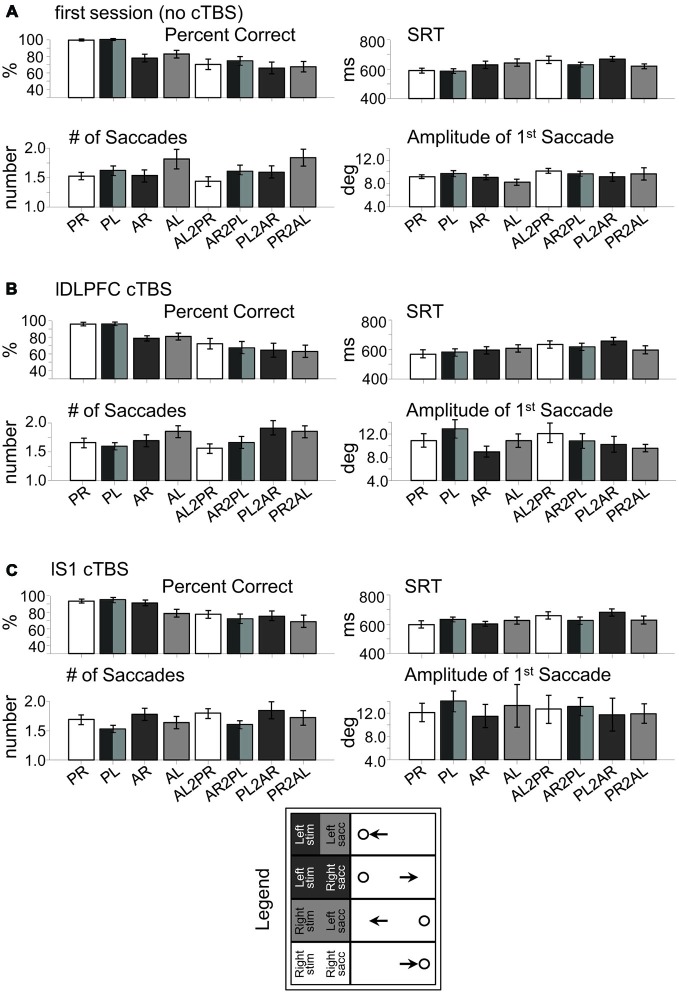
**Raw behavioral data in lDLPFC study. (A)** Session 1 (no cTBS). **(B)** lDLPFC cTBS. **(C)** lS1 cTBS. Conventions are as in Figure 4.

There were eight trial types of interest consisting of: correct pro, anti, pro-to-anti and anti-to-pro trials, with left/right direction considered separately (Figures 3–6). Thus, a 2 × 2 × 2 × 2 (four-way) repeated measures ANOVA (Figures 3, 3, 5) between rS1 and rFEF, or lS1 and lDLPFC stimulation were conducted across subjects with the eight response types divided by factors of Stimulus Location (right or left), Initial Task instruction (pro or anti), Switch Condition (non-switch or switch) and Site of cTBS (oculomotor site or S1). The expectation-maximization (EM) method for missing cells was employed using SPSS Statistics 21 (IBM) satisfying Little’s Missing Completely At Random (MCAR) test. To aid in illustration of the four-way ANOVA, Figures 3, 3, 5 plot the rFEF-rS1 or lDPFC-lS1 *differences*. One-sample *t*-tests were conducted on these differences and are also illustrated in Figures 3, 3, 5. Also, as our main interest is the cTBS effects, we report the main effects or interactions involving Site.

## Results

### rFEF Stimulation

Figure 3 shows these effects as a subtraction, with the positive axis corresponding to greater measurement values following rFEF cTBS, and the negative axis corresponding to greater measurement values following rS1 cTBS. Figure 3 displays the significant results from one-sample *t*-tests for each trial type (illustrating a significant difference from zero for the rFEF-rS1 cTBS subtraction), and Figure 4 shows the raw data across each parameter for each session separately, including the first (no-cTBS) day.

For performance accuracy (Percent Correct; Figure 3A), there were no significant interactions or main effects (*p*s > 0.17), meaning that rFEF cTBS did not affect performance accuracy in terms of executing a saccade in the proper direction.

For SRT (Figure 3B), there was a significant Site × Switch Condition interaction, *F*_(1, 13)_ = 9.88, *p* < 0.01, driven by greater SRT difference between rFEF cTBS and rS1 cTBS during switch trials compared to non-switch trials.

For the number of saccades made by each subject (Figure 3C), there was a main effect of Site, *F*_(1, 14)_ = 19.96, *p* < 0.001, and there was a significant interaction between Site, Initial Task and Switch Condition, *F*_(1, 14)_ = 8.72, *p* < 0.05. This latter result was because there were a greater number of saccades after rFEF cTBS when an anti-saccade was executed (i.e., non-switch anti-saccade trials and pro-to-anti-saccade switch trials) rather than when a pro-saccade was executed (i.e., pro-saccade and anti-to-pro-saccade). The main effect of Site reflects the fact that there was an overall increase in the number of saccades after rFEF cTBS compared to rS1 cTBS (Figure 3C).

Finally, for saccade amplitude, there was also a main effect of Site *F*_(1, 14)_ = 7.12, *p* < 0.05, and there was a significant Site × Initial Task × Switch Condition interaction, *F*_(1, 14)_ = 4.98, *p* < 0.05, as there was an overall reduced amplitude for anti-saccades compared to pro-saccades after rFEF cTBS (Figure 3D).

### Summary

Following cTBS to rFEF as compared to rS1, there were a greater number of saccades before reaching stable position and reduced anti-saccade amplitude. These deficits are consistent with anti-saccade hypometria. There were no significant increases in pro-saccade errors on anti-saccade trials, but there was an increase in switch trial reaction time.

### lDLPFC Stimulation

Figure 5 shows the differences in saccade behavior following cTBS to left DLPFC compared to left S1. Conventions for displaying the results and the analysis are the same as for rFEF stimulation. Figure 5 also shows the results that reached significance from one-sample *t*-tests of the lDLPFC-lS1 difference measures, while Figure 6 shows the behavior for each parameter on each session.

For performance (Figure 5A), there was a significant Site × Stimulus Location interaction, *F*_(1, 14)_ = 4.83, *p* < 0.05, and a significant Site × Initial Task × Switch Condition interaction, *F*_(1, 14)_ = 6.46, *p* < 0.05. These combined effects were driven by impaired performance following rFEF cTBS during non-switch anti-right trials and pro-left-to-anti-right switch trials.

For the remaining parameters: SRT (Figure 5B), the number of saccades (Figure 5C) and saccade amplitude (Figure 5D), there were no significant effects (*p*s > 0.06).

### Summary

Following cTBS to lDLPFC as compared to lS1, there were lateralized deficits during anti-saccades, such that subjects executed more pro-saccade errors to the left, on trials requiring an anti-saccade to the right. Thus, lDLPFC cTBS impaired contralateral anti-saccade performance.

## Discussion

We observed dissociable effects from inhibitory cTBS to the FEF or DLPFC on saccade behaviors. cTBS to left DLPFC caused an increase in pro-saccade errors on contralateral anti-saccade trials, suggesting DLPFC is critical for executive control of saccades. These impairments were not observed after right FEF cTBS; instead, we observed that right FEF cTBS increased saccade steps and caused anti-saccade hypometria, suggesting that FEF is critical for visuo-motor processing for saccades. Lateralization effects are discussed in the following sections, and suggest that the directional specificity is in line with ipsi/contralateral properties of the oculomotor system.

### The Effect of Altered FEF Function on Saccade Behavior

Given knowledge of different FEF neural subtypes, and our understanding of their correlates to saccade behavior, FEF has been an appropriate candidate to test if inhibitory TMS will induce changes to visuo-motor processing, and also executive control. It is known that some FEF neurons code for the motor goal of saccades, while others process visual and visuomotor information, with a dominance for contralateral processing (Bruce and Goldberg, 1985; Schlag-Rey et al., 1992; Schall, 2002; Sato and Schall, 2003; Schall et al., 2011). For an anti-saccade, subjects must invert a visual vector coding stimulus position into a motor vector to program a saccade to the mirror location (Collins et al., 2008), and this vector inversion process is accomplished in part by FEF and also by the parietal eye fields (PEF) in the intraparietal sulcus region (Zhang and Barash, 2000; Medendorp et al., 2005; Moon et al., 2007). Here, we observed deficits in the vector inversion process, as anti-saccades became hypometric and the number of anti-saccade “steps” increased, confirming that FEF is important to the vector inversion process. Recently, Jaun-Frutiger et al. (2013) observed that cTBS to right FEF resulted in hypometric rightwards, and not leftwards anti-saccades, and thus proposed cTBS to FEF impaired inverting the visual vector, which is the component related to the computed spatial distance (as opposed to a motor vector, which is a saccade program). While the actual inversion process itself is not fully understood, it has been demonstrated that anti-saccade amplitude is more closely linked to developing a visual vector (Collins et al., 2008). We did not observe a statistical difference between left-wards and right-wards anti-saccades, though the trends are in agreement with this finding (Figures 3C,D), particularly for non-switch anti-saccades. We speculate that switch trials complicate this interpretation, as a developed saccade program (a motor vector) may be switched with respect to being in line with the visual vector, when the instruction changes. Thus, we can conclude that rFEF cTBS impaired the vector inversion process in anti-saccade generation, but cannot say here whether this is specifically related to impairing the visual vector.

In addition to FEF saccade neurons, fixation neurons (which are tonically active during fixation) are also present. So how can we reconcile the fact that we did not observe deficits in executive control after rFEF cTBS, given our knowledge of the activity profiles of fixation and saccade neurons during anti-saccade tasks (Everling and Munoz, 2000; Munoz and Everling, 2004), and during other studies involving saccade suppression (Boucher et al., 2007; Ramakrishnan et al., 2012; Schall and Godlove, 2012)? It is possible that FEF activity observed in these studies during anti-saccade tasks reflects input from higher regions, such as the DLPFC, which cannot be ruled out in any physiological study (e.g., single-unit recording or fMRI), as even the output signals measured in individual FEF neurons can be shaped by incoming signals in addition to local neural processes. Fixation and saccade neurons are also present in the superior colliculus (SC) with similar discharge patterns on anti-saccade trials to that observed in FEF (Everling et al., 1998, 1999; Munoz and Everling, 2004; Boucher et al., 2007). These SC neurons have been demonstrated to receive task-related signals from DLPFC (Johnston and Everling, 2006), and to our knowledge, no such study has been done linking DLPFC neurons to FEF activity. The basal ganglia (BG) is also proposed to be involved in saccade suppression and anti-saccade facilitation via influences on the SC, as well as on thalamo-cortical loops (Munoz and Everling, 2004; Watanabe and Munoz, 2011). It is therefore possible that FEF neurons carry executive control signals in the relative activation profiles of fixation and saccade neurons, but are not their source. Similarly, it is possible that other oculomotor structures that also carry these signals can maintain the functions necessary for performing a correct saccade in voluntary tasks such as this, even when FEF’s contribution is impaired. While there are reported findings in the literature that do suggest a direct role of FEF in inhibiting reflexive saccades, the results are inconclusive. The results do however support the role of FEF in being critical to programming a voluntary saccade to a particular spatial location.

Some patients with frontal lesions encompassing FEF were shown to have deficits inhibiting pro-saccades in an anti-saccade task (Guitton et al., 1985), and more recently four patients with FEF lesions also exhibited deficits in inhibiting contralateral reflexive saccades (Van der Stigchel et al., 2012); however, two of these patients had lesions that involved DLPFC. In another study of a patient with a highly circumscribed left FEF lesion, there were no deficits in inhibiting reflexive saccades, but there were deficits in saccade amplitudes (Gaymard et al., 1999). On the other hand, a single TMS pulse to FEF, 100 ms after stimulus appearance, was shown to increase pro-saccade errors during an anti-saccade task (Terao et al., 1998). However, note that TMS pulses *during* anti-saccade generation perturbs an evolving saccade program, which engages FEF saccade neurons when voluntary signals must outcompete more automatic signals (Munoz and Everling, 2004). Other studies using TMS to FEF, however, have not reported changes in error rates on anti-saccade trials, though they have found increased reaction times when the pulses were applied at critical time periods during saccade programming (Müri et al., 1991; Olk et al., 2006), or at the end the preparatory period (Nagel et al., 2008). Likewise, we also found a main effect of increased reaction times on switch trials compared to non-switch trials after rFEF cTBS, and another FEF cTBS study which used a complex paradigm (subjects made pro- or anti-saccades to an oddball stimulus based on the instructional cue) also found increases in reaction times for both pro- and anti-saccade responses (Liu et al., 2011). Given these previous findings, and the results of this study, we propose that FEF is not critical to executive control, but is part of a network that carries task set signals, and it is obviously important to programming a voluntary saccade; impairments are particularly detectible as increased reactions times, when the demands for voluntary saccade programming are high, such as when one must suddenly generate a saccade to a different goal on switch trials. This conclusion also does not rule out a role of FEF in other top-down signals, as FEF has been demonstrated to modulate activity in early visual regions (Moore and Armstrong, 2003; Ekstrom et al., 2009; Ruff et al., 2009).

### The Effect of Altered DLPFC Function on Saccade Behavior

In patient studies, lesions to DLPFC have resulted in increased pro-saccade errors on anti-saccade trials (Guitton et al., 1985; Pierrot-Deseilligny et al., 1991, 2003; Ploner et al., 2005), suggesting a role of DLPFC in reflexive saccade suppression. However, it has been difficult to dissociate a suppression role of DLPFC from a role in task set establishment, which could also be observed as an increase in pro-saccades errors following DLPFC lesion; this is because anti-saccade task set signals are needed as a bias the oculomotor system against the more automatic pro-saccade behavior. DLPFC neurons recorded in monkeys show instruction-related activity, with separate neurons signaling the anti-saccade instruction and others the pro-saccade instruction (Everling and DeSouza, 2005), suggesting that DLPFC neurons represent task set. Likewise, other human and monkey studies have found “preparatory” signals during pro- or anti- instruction periods in DLPFC (as well as in FEF; Everling and Munoz, 2000; Connolly et al., 2002; DeSouza et al., 2003; Everling and DeSouza, 2005; Ford et al., 2005; Brown et al., 2007; Cameron et al., 2012). It has been found however that it is the DLPFC neurons which signal *anti-saccade* task set that project to the SC (Johnston and Everling, 2006; Johnston et al., 2009), and these neurons influence saccade neuron, not fixation neuron, activity. This finding is more consistent with the notion that DLPFC neurons code task set signals rather than suppression signals (Everling and Johnston, 2013; Johnston et al., 2014). Pro-saccade errors, therefore, can be explained by disruption to task set signals that did not bias the balance between anti- and pro-saccade signals. In the present study, we did not observe significant effects from cTBS to DLPFC on reaction time. However, the observation that non-switch pro-left trials were facilitated in terms of a faster SRT (Figure 5B) after lDLPFC cTBS is consistent with an interpretation that there was a disruption in signals that would normally bias against pro-saccade execution. Because we employed a task switching design, there is always the possibility of anti-saccade bias signals being present, as even during pro-saccade instruction, subjects may have to produce an anti-saccade subsequently. Thus, competition from these anti-saccade bias signals may have been reduced after lDLPFC cTBS, resulting in faster reaction times. We note that in the task switching design, subjects typically exhibit increased reaction times even on non-switch pro-saccade trials (Figure 2) in comparison to what is typically observed (on the order of 150–300 ms; see also Cameron et al., 2010). However, whether these bias signals could represent response suppression rather than anti-saccade task set still needs to be resolved. For instance, in a previous TMS study, a single pulse to DLPFC 100 ms before stimulus onset (and not after) increased pro-saccade errors in an anti-saccade task, and this was proposed to be due to impaired inhibitory signals from DLPFC (Nyffeler et al., 2007). Note that these findings could be explained as a deficit in anti-saccade task set. In another study, a single TMS pulse to left DLPFC at the end of a preparatory period increased both pro- or anti-saccade reaction times (they did not find increased error rates; Nagel et al., 2008), and the authors interpreted this as a disruption to “preparatory” set, which is sensible, because if this pulse impaired suppression signals, subjects should have been faster, at least for pro-saccades.

The laterality effects observed in the current study also indicate more of an effect on saccade bias signals, to the contralateral side, than to a specific effect of left DLPFC in executive control in general. It has been shown that patients with left DLPFC lesions display deficits in task set establishment, whereas patients with right prefrontal lesions display deficits in task monitoring (Stuss and Alexander, 2007; Stuss, 2011), suggesting potentially different roles of left and right DLFPC in executive control. However, our observation of greater pro-saccade errors on anti-right and pro-left-to-anti-right trials (rather than increased errors independent of stimulus location), indicates mainly a spatial-specific effect, consistent with the lateralization of the oculomotor system in terms of saccade programming. DLPFC neurons have receptive/response fields with a contralateral bias (across the population) in the delay-period in working memory tasks (Funahashi et al., 1989; Ikkai and Curtis, 2011). Secondly, cooling unilateral DLPFC lead to reduced saccade neuronal activity in the ipsilateral SC, and increased activity in the contralateral SC, whereas cooling both DLPFCs affected SC saccade neurons bilaterally (Koval et al., 2011). Thus, observed laterality effects align with contra/ipsi aspects of saccade programming. However, such effects have not always been found consistently in lesion or TMS studies, as some have produced bilateral effects over *left or right* DLPFC: a single TMS pulse to right DLPFC 100 ms before stimulus onset resulted in bilateral increases in pro-saccade errors in an anti-saccade task (Nyffeler et al., 2007), and “intermittent” TBS (which has excitatory rather than inhibitory effects; Huang et al., 2005) over left DLPFC resulted in bilateral reduction in pro-saccade errors in patients with bipolar disorder (Beynel et al., 2014). Pierrot-Deseilligny et al. (2003) also observed increased pro-saccade errors, bilaterally, on anti-saccade trials with left (two patients) or right (one patient) DLPFC lesions. In sum, while consistent laterality effects are not always observed, there is convincing evidence across previous studies that DLPFC is critical to executive control in saccade tasks.

Finally, because of the spatial properties of the DLPFC neurons, particularly important in working memory tasks, we also acknowledge a possible contribution of DLPFC to saccade programming in terms of metrics. Indeed, TMS to left and right DLPFC has been shown to affect endpoint accuracy in memory-saccades (Brandt et al., 1998). However, here, in a non-memory design, we did not find any main effects or interactions after lDLPFC cTBS on the number of saccades or the saccade amplitude, suggesting that any potential role of DLPFC is less than that of FEF.

**Table 1:** **Average (mm) ± SD MNI coordinates for cortical oculomtor regions**.

	*X*	*Y*	*Z*
**FEF study**
cTBS sites
rFEF	30 ± 6	−6 ± 4	56 ± 6
rS1	9 ± 2	−39 ± 5	79 ± 1

lFEF	−27 ± 6	−8 ± 3	54 ± 6
rPEF	20 ± 6	−67 ± 8	58 ± 8
lPEF	−20 ± 8	−65 ± 8	58 ± 7
rDLPFC	36 ± 7	43 ± 7	29 ± 5
lDLPFC	−36 ± 6	39 ± 4	29 ± 6
SEF	2 ± 8	0 ± 9	65 ± 4
**DLPFC study**

lDLPFC	−38 ± 5	40 ± 4	28 ± 6
lS1	−8 ± 2	−43 ± 3	77 ± 2

rFEF	30 ± 6	−5 ± 5	57 ± 5
lFEF	−28 ± 6	−9 ± 3	57 ± 8
rPEF	25 ± 6	−68 ± 8	56 ± 6
lPEF	−23 ± 7	−67 ± 7	57 ± 5
rDLPFC	38 ± 5	42 ± 5	27 ± 5
SEF	1 ± 6	5 ± 10	62 ± 6

*DLPFC, dorsolateral prefrontal cortex; FEF, frontal eye fields; l, left; MNI, Montreal Neurological Institute (stereotactic atlas); PEF, parietal eye fields; r, right; S1, primary somatosensory cortex; SD, standard deviation; SEF, supplementary eye fields*.

### Study Limitations

#### Eye Tracking and Behavior

We performed all experimental sessions in the fMRI environment in order to characterize neural activation patterns (in a companion study). Eye-tracking calibration was not possible during scanning due to constraints on time during which cTBS exhibits its effect. However, we controlled for directional differences in amplitudes (i.e., leftward and rightward responses each consisted of four different response types). Secondly, while there were slightly different saccade amplitudes (12 and 15^°^) to the targets across each study, these eccentricities fall within the typical range of 10–15^°^ classified as “large” in many human fMRI studies of spatial mapping (Tootell et al., 1998; Sereno et al., 2001; Schluppeck et al., 2005; Swisher et al., 2007). Differences in a 10–15^°^ range are not thought to be fundamentally different in terms of saccade visuo-motor processing (at least at the cortical representational level), where differences are usually found when compared to small saccades (e.g., <4^°^; Silver et al., 2005; Leoné et al., 2014). Note also that Jaun-Frutiger et al. (2013); observed their effects on vector inversion from FEF cTBS collapsed across a range of 8–16^°^ targets. Thus, it is unlikely that executive control (measured by a gross movement in the wrong direction) or the metrics and number of step saccades would be different between a 12 and 15^°^ saccade, especially given that the effects of cTBS to FEF or DLPFC were compared to a control stimulation site.

We also note that the reaction times in this study are indicative of a waiting strategy in the participants, which was also found in Cameron et al. (2007); Cameron et al. (2009); Cameron et al. (2010); specifically, the mean latencies are indeed longer than what is typical in pro- and anti-saccade tasks (on the order of 150–300 ms) that do not employ switching. However, the key factor in these studies is the relative difference in automaticity; pro-saccades are more automatic than anti-saccades, even if an uncertainty of task switching induces a waiting strategy. Note that even a pro-saccade/anti-saccade interleaved design will increase the latencies as compared to a blocked design (Cameron et al., 2010), because there cannot be foreknowledge about which task will be required on the current trial, and because of additional costs associated with switching tasks across trials. Importantly, this does not change the relative difference in automaticity between the tasks, and interleaved designs are used quite frequently despite slower reaction times overall.

Finally, we acknowledge that subjects produced hypometric saccades, even in the no-cTBS session (Figures 4A, 4A, 6A). Saccade amplitude was not examined in our previous studies that used this switching paradigm (Cameron et al., 2007, 2009; Cameron et al., 2010), though we do find that participants often make hypometric saccades, at least in generating a saccade step. We suspect that like the increased latencies, this may relate to the uncertainty of the task, in particular, to the uncertainty in response direction. It was previously shown that uncertainty in target eccentricity across trials results in subjects producing markedly hypometric anti-saccades (that were in fact an average of the target eccentricities), though pro-saccades were of appropriate gain (Dafoe et al., 2007). Here, the possibility of switching task may have resulted in response uncertainty and produced hypometric saccades. The important point here is that this was independent of the cTBS comparisons, though future studies could examine more thoroughly how task uncertainty interacts with saccade metrics.

#### Continuous Theta-Burst Stimulation

Though each study employed separate groups of participants, the main comparisons (i.e., between cTBS to the oculomotor site and cTBS to S1) were conducted in the same subjects in a counterbalanced fashion. Thus, the comparisons controlled for non-specific effects of cTBS, allowing us to determine the specificity of the “virtual lesion” effects, where an interpretation between the effects of FEF cTBS and DLPFC cTBS in different subjects is similar to comparing FEF and DLPFC lesions in patient studies. However, we do acknowledge the potential for non-specific effects from the S1 cTBS. It is important to first note that only the S1, and FEF or DLPFC cTBS sessions were counterbalanced, so direct comparisons between the S1 cTBS condition and the first session (no cTBS) are inappropriate. Nevertheless, it is possible that there were modulatory effects on behavior from S1 cTBS (Figures 4, 4, 6). We cannot conclusively rule out such effects, but it is unlikely that the effects of cTBS to S1 could be *greater* than the effects of cTBS to the oculomotor regions themselves for three reasons. First, we confirmed in our fMRI analysis that there was no significant saccade-related activations in these S1 regions of interest, but that were present in the lDLFPC and rFEF regions, as well as in other nearby cortical oculomotor regions (i.e., the supplementary eye-fields, and “parietal eye fields” in the intraparietal sulcus). Offline repetitive TMS protocols are all best understood as having influences on synaptic plasticity (Ziemann and Siebner, 2008; Di Lazzaro et al., 2010), so it is unlikely that a region not involved in the task would produce an effect on behavior, even if local synaptic processes were changed. Second, the distance between these S1 sites and the right FEF and left DLPFC exceeded a reasonable assumption for the required distance to dissociate TMS effects between two regions (i.e., twice a 6–8 mm radius; Wagner et al., 2009). Shown in Table 1; we also assessed the distance from the S1 sites and other cortical oculomotor structures (as revealed by the same fMRI method for localizing rFEF and lDLPFC): the left FEF, midline supplementary eye fields, and right and left PEF, and all were greater than this distance (min Euclidian distance between any of the cTBS sites and other regions: 30 mm). Finally, while it is certain that the TMS-induced electric field passes through neighboring gray matter and white-matter tracts (De Geeter et al., 2015), inhibitory cTBS protocols are best understood as influencing the so-called “indirect waves”, which result at cortico-cortico synapses (Di Lazzaro et al., 2010), and any non-specific influence of the electric field would penetrate neighboring gray and white matter around the oculomotor stimulation sites as well. Therefore, the main possibility is that there was a practice/placebo effect of cTBS from S1 cTBS itself, but we acknowledge the possibility of some unknown influence of the electric field on neighboring tissue that would be different depending on the stimulation site.

## Funding

This work was supported by the National Institutes of Health (MH63901 to MD) and a Canadian Institutes of Health Research Postdoctoral Fellowship to IGMC.

## Conflict of Interest Statement

The authors declare that the research was conducted in the absence of any commercial or financial relationships that could be construed as a potential conflict of interest.
